# PARPs, PAR and NAD Metabolism and Their Inhibitors in Cancer

**DOI:** 10.3390/cancers12123494

**Published:** 2020-11-24

**Authors:** Nicola Curtin, Péter Bai

**Affiliations:** 1 Newcastle University Centre for Cancer, Translational and Clinical Research Institute, Faculty of Medical Sciences, Newcastle University, Newcastle upon Tyne NE2 4HH, UK; 2 Department of Medical Chemistry, Faculty of Medicine, University of Debrecen, 4032 Debrecen, Hungary; 3 MTA-DE Lendület Laboratory of Cellular Metabolism, 4032 Debrecen, Hungary; 4 Research Center for Molecular Medicine, Faculty of Medicine, University of Debrecen, 4032 Debrecen, Hungary

The role of poly(ADP-ribose) polymerase-1 (PARP1) in DNA repair and as a potential target for anticancer therapy has been under investigation for more than 50 years. The field has expanded over the decades to include not only a family of ADP-ribosylating enzymes (PARPs/ARTDs), but also interacting and polymer-degrading proteins. In this special issue of *Cancers* primary research articles and reviews describe various aspects of PARP biology along with therapeutic targeting. Some historical perspective is reviewed along with the development of the PARP inhibitor (PARPi), rucaparib/Rubraca^®^, including the identification of the synthetic lethality with homologous recombination repair (e.g., BRCA) defects [1]. Further consideration of the mode of action of several PARPi and their interaction with cytotoxic chemotherapy is described by Min and Im [2], and the evidence for a unique mode-of-action of another PARPi, PJ34 [3] is summarized. Several other aspects of PARPi therapy are described, including research indicating the therapeutic potential of the PARPi, olaparib, as a single agent in myelodysplastic syndromes, not only through cytotoxic and cytostatic effects but also through the induction of differentiation [4], along with the augmentation of UVB-induced DNA damage and mitochondrial alterations resulting in reduced proliferation and viability of keratinocytes [5]. On the other side of the coin, deficiency of the enzyme that degrades the PARP-generated ADP-ribose polymers, PARG, in ES cells resulted delayed tumour growth when they were implanted SC and increased antitumour activity of X-rays [6].

Moving PARPi therapy beyond BRCA mutated cancers requires the use of biomarkers to predict PARPi sensitivity as reviewed by Singh et al. [7]. The role of defects in the G1 cell cycle checkpoint signaling kinase, ATM, as a determinant of sensitivity to PARPi was reviewed, with the finding that PARPi alone are cytostatic in ATM defective cancer cells but require the addition of an inhibitor of the S/G2 cell cycle checkpoint kinase, ATR, to induce cell death [8]. An investigation of the synergy between the PARPi, olaparib, and the ATR inhibitor VE-821 in a panel of neuroblastoma cells, revealed that it was independent of MYCN or ATM status in these cells [9] and similar studies identified that the synergy between an inhibitor of the S/G2 cell cycle checkpoint kinase, CHK1, and the PARPi, rucaparib was largely through the impairment of homologous recombination repair by the CHK1 inhibitor [10]. Depletion of p60/150 CAF-1 also impaired homologous recombination repair thereby inducing sensitivity to PARPi and irradiation in head and neck cancers with therapeutic implications [11]. Interestingly, depletion of NMNAT1, which is involved in the synthesis of NAD+, PARP’s substrate, induced DNA damage and sensitized cells to cisplatin but exhibited redundancy with PARPi in this respect [12]. Inhibition of EGFR or Syk (which mediates EGFR signaling) was synergistically cytotoxic in combination with olaparib in squamous cell carcinoma cells suggesting therapeutic potential [13].

Of course, resistance to PARPi is an emerging problem clinically and the role of the PI3K-AKT pathway in protection from PARPi-induced cytotoxicity and its significance in shock, inflammation, ischemia-reperfusion injury and cancer is reviewed by Gallyas et al. [14]. Interestingly, gastric cancer cell lines made resistant to olaparib were found to be cross-resistant to cisplatin, but had increased sensitivity to irinotecan due to upregulation of TOP1 and TDP1, which has therapeutic implications for patients who develop PARPi resistance [15].

PARP has been investigated as a regulator of transcription and key roles regarding the role of PARP both in nucleolar function in relation to cancer biology and the role PARylation plays in the regulation of transcription were reviewed [16,17]. With original research showing that PARylation activates the histone acetyl transferase, EP300, contributing to its regulation of transcription of DNA repair and proliferation genes [18].

An important factor in cancer biology is the tumour microenvironment and the interaction between PARP and key features of the tumour microenvironment such as autophagy, hypoxia and angiogenesis was reviewed [19]. There is significant interest in the immune microenvironment and the roles of both PARP1 and PARP2 in modulating both the innate and adaptive immune system was reviewed by Yelamos et al. [20], with the therapeutic potential of the combination of PARPi with immune checkpoint inhibitors, including translational and clinical studies were reviewed by Peyraud and Italiano [21].

Feijs and colleagues [22] reviewed the roles of ADP-ribosyl hydrolases, also involved in the degradation of ADP-ribose and poly(ADP-ribose) chains, MACROD1, MACROD2 and TARG1 in carcinogenesis. The role of ARH1, another degradatory enzyme, was reviewed by Ishiwata-Endo and colleagues [23] in cancer and non-cancer diseases.

The roles for poly(ADP-ribosyl)ation in biochemistry, cell biology, physiology and pathophysiology are rapidly expanding, the findings discussed in the “PARPs, PAR and NAD Metabolism and Their Inhibitors in Cancer” special issue of Cancers will surely provide a better understanding of these processes and widen the scope of our appreciation of poly(ADP-ribosyl)ation.
